# Guillain–Barre syndrome and link with COVID-19 infection and vaccination: a review of literature

**DOI:** 10.3389/fneur.2024.1396642

**Published:** 2024-06-05

**Authors:** Vijaya Lakshmi Valaparla, Schweta P. Rane, Chilvana Patel, Xiangping Li

**Affiliations:** University of Texas Medical Branch at Galveston, Galveston, TX, United States

**Keywords:** Guillain–Barre Syndrome (GBS), COVID-19, COVID-19 vaccine, peripheral nervous system, autoimmune disease

## Abstract

**Background:**

Guillain–Barré syndrome (GBS) is an autoimmune disease associated with significant morbidity. A wide variety of infectious and non-infectious triggers have been identified to be associated with GBS. COVID-19 has gained attention in recent years for its role in GBS pathogenesis. Our study aims to review the literature on GBS and its epidemiological and pathophysiological association with COVID-19.

**Description:**

Recent literature on GBS associated with COVID-19 infections, such as case reports, case series, systematic reviews, and large-scale epidemiological studies, were reviewed. We also reviewed studies that included vaccines against COVID-19 in association with GBS. Studies that focused on understanding the pathobiology of GBS and its association with infectious agents including COVID-19 were reviewed.

**Conclusion:**

Despite a lack of consensus, GBS is strongly associated with COVID-19 infection. The exact pathophysiological mechanism regarding COVID-19 as a causative agent of GBS is unknown. Mechanisms, such as the proinflammatory state, triggering of autoimmunity, and direct viral invasion, are postulated and remain to be investigated. Adenovirus vector vaccines are most likely associated with GBS, and the consensual reports clearly suggest mRNA vaccines are associated with low risk and may be protective against GBS by reducing the risk of COVID-19 infection.

## Introduction

Guillain–Barré syndrome (GBS) is an autoimmune disease characterized by progressive limb weakness, sensory deficits, cranial nerve involvement, tendon areflexia, and cerebrospinal fluid (CSF) albumin cytological dissociation with three major pathophysiological phenotypes: acute inflammatory demyelinating polyneuropathy (AIDP), acute motor axonal polyneuropathy (AMAN), and acute motor and sensory axonal neuropathy (AMSAN) (1). It was first described in 1916 by Guillain, Barre, and Strohl when they reported two soldiers with acute flaccid paralysis with high cerebrospinal fluid protein levels and normal cell counts (2). GBS is the most common cause of acute flaccid paralysis in the United States and is almost always preceded by an antecedent infection, most commonly by *Campylobacter jejuni* (*C. jejuni*); however, over the last century, many more associations have come to light, the most recent being the novel SARS-CoV-2 virus or the COVID-19 pandemic.

The annual global incidence of GBS as of 2020 was 0.6–4 /100,000 person-years (3). With the advent of the pandemic, over 200 cases of GBS following COVID-19 infection have been reported from at least 23 countries, revealing an overall reported prevalence of at least 15/100,000 population-years among the COVID-19 patients (4). The incidence of GBS was noted to have significantly increased during the pandemic when compared with the same months during the non-pandemic years, thus suggesting a pathogenic association and that COVID-19-related GBS is more severe compared to non-COVID-related GBS (4–6).

GBS is usually preceded by infection or immune stimulation, which induces an aberrant autoimmune response driven by molecular mimicry of microbial surface molecules that target the axolemma/myelin of peripheral nerves and their spinal roots (6). The molecular mimics are glycans expressed on lipooligosaccharides (LOS) of preceding infectious organisms that can induce humoral-mediated antibody responses independent of T cells. Macrophages attack the myelin sheath by penetrating the basement membrane around nerve fibers and strip normal myelin sheath away from Schwann cell bodies and off the axon. This disruption of the anatomical and physiological integrity of exposed nerve membranes in nerve terminals and nodes of Ranvier causes a nerve conduction blockade that is either reversible or, in severe cases, results in widespread axonal degeneration with poor recovery (7).

Some antibody specificities are associated with specific GBS subtypes, and the related neurological deficits reflect the distribution of different gangliosides (individually or in combination) on human peripheral nerves. For example, *C. jejuni* infections are predominantly, but not exclusively, related to the AMAN or pure motor subtype of GBS; have serum antibodies against GM1a, GM1b, GD1a, and GalNAc-GD1a gangliosides; and are of the subclass IgG1 and IgG3 (8, 9). Patients with Miller Fisher syndrome (MFS) or MFS–GBS overlap syndrome frequently have antibodies against GD1b, GD3, GT1a, and GQ1b gangliosides (10). Antibodies against proteins in the specialized domains at the nodes of Ranvier, including gliomedin, contactin, TAG-1, moesin, and neurofascin, have been identified in addition to anti-GM2 antibodies in association with preceding cytomegalovirus (CMV) infection (11). Other infections associated with Guillain–Barré syndrome are Epstein–Barr virus, Zika virus, influenza A virus, *Mycoplasma pneumoniae*, hepatitis E, hepatitis B, human immunodeficiency virus (HIV), and *Haemophilus influenzae* (12). A limited number of cases have also shown T-cell responses (in addition to B-cell) to compact myelin proteins, including P0, P2, and PMP22, and gangliosides expressed in glial membranes at the nodes of Ranvier (13). However, the interplay between microbial and host factors that dictate it and how the immune response is shifted toward unwanted autoreactivity are still not well understood.

GBS has been reported in response to other noninfectious triggers such as recent stress, surgeries, trauma, pregnancy, and the postpartum period (14). GBS is also associated with neoplastic conditions such as lymphoma via direct invasion of the nerve trunks with lymphoma cells (neurolymphomatosis), vascular insult causing nerve infarct, or by causing an inflammatory cascade leading to damage to the axon (15). Anticancer therapies such as immune checkpoint inhibitors have also been associated with GBS (16). Although the role of autoimmunity is a likely mediating factor, the exact mechanisms by which these triggers lead to disease manifestations remain unclear.

For the current review, a literature search was conducted in the PubMed Central, Google Scholar, and ScienceDirect databases. The keywords included were “COVID 19” or “SARS CoV-2” or “Coronavirus,” “Guillain Barre Syndrome” or “Acute Inflammatory Demyelinating Polyneuropathy” or “GBS” or “AIDP” or “Miller Fisher Syndrome,” and “COVID 19 Vaccination.” We included large cohort studies, systematic reviews, literature reviews, and case series that discussed the areas of interest.

## Implications of COVID-19 on the neuromuscular system

SARS-CoV-2 or COVID-19, although predominantly a respiratory pathogen, involves multiple systems, including the nervous system. While disease manifestations in the central nervous system such as seizures, encephalopathy, stroke, and myelitis gained attention earlier on during the pandemic, involvement of the peripheral nervous system has been extensively reported (17–19). Based on the anatomical targets down the neuroaxis, these manifestations can be divided into three categories: nerve-related, neuromuscular junction-related, and muscle-related (19).

Nerve involvement can manifest as Guillain–Barre Syndrome (GBS), cranial neuropathies, plexopathies, and critical illness neuropathies (19). GBS and its variants have been, by far, the most extensively reported entities among the nerve-related manifestations of COVID-19.

## Does COVID-19 infection increase the risk of GBS?

The incidence of GBS in the post-infection period of COVID-19 has received significant attention in the early pandemic era. Ever since the first case of COVID-19-associated GBS was reported from Wuhan, China, multiple subsequent studies reported an increase in GBS cases in association with COVID-19 infection (4, 20, 21). Thus, GBS was thought to be a para-infectious process of COVID-19, with a recent review reporting over 200 cases of GBS associated with SARS-CoV-2 infection across nations worldwide (22). A large-scale population-based study from Israel has reported six times increased risk of GBS in those with COVID-19 infections, in comparison to the control group (23). These initial studies followed by subsequent systematic reviews of GBS associated with COVID-19 suggested a possible causative role of COVID-19 and a potential risk factor for GBS. A few other studies including a large United Kingdom (UK)-based epidemiological study found that there were no epidemiological or phenotypic associations between GBS and COVID-19 infection, leaving this hypothesis a questionable entity (24–27). The results from epidemiological studies based on the UK National Immunoglobulin Database (27) suggest that the overall incidence of GBS during the pandemic era has shown a downward trend. These results should be interpreted cautiously as factors such as social distancing and decreased public dining have also resulted in decreased incidence of seasonal flu and *C. jejuni* infection rates. These factors might have helped decrease the incidence of other known risk factors of GBS.

## Role of vaccines against COVID-19

GBS associated with flu vaccination has been in discussion since the introduction of mass vaccination campaigns against swine flu in 1976 (28). Despite the initial observation that vaccination increased the risk of GBS, the exact pathophysiological mechanism for mediating this association was unclear. An association between seasonal flu vaccines and GBS, although noted from case studies and anecdotal reports, was not established based on larger epidemiological studies (29).

The debate regarding vaccines against COVID-19 that cause GBS came into existence with mass vaccination campaigns that were launched to curtail the COVID-19 pandemic. Different types of vaccines, including mRNA, viral vector, and killed virus vaccines, have been developed. A recently published systematic review found the prevalence of COVID-19 vaccination-related GBS to be 8.1 per 1000,000 vaccinations (30). The same study also found that this increased risk was associated with adenovirus vector vaccines more so than mRNA vaccines (30, 31). Similar results were published from the data obtained from the Vaccine Adverse Event Reporting System (VAERS), which is a vaccine safety surveillance system co-administered by the Centers for Disease Control (CDC) and the Food and Drug Administration (FDA) (31). This study found that the risk of GBS with adenovirus vector vaccination was 8–10 times higher than that with mRNA vaccines. The risk of GBS in those receiving adenovirus vector vaccination was 2–3 times higher than that in the general population (31). Few other studies have safely concluded that mRNA vaccines reduced the risk of GBS by providing protection against COVID-19 infection (23). Along the same lines, recent studies strongly suggest that the risk of GBS with vaccination remains far less compared to that with COVID-19 infection itself, and the benefit of vaccinations outweighs the risk (31, 32).

Apart from intramuscular administration, vaccines are also studied and developed for intradermal and intranasal administration (33, 34). The majority of these are adenovirus vector-based vaccines based on WHO data (35). It is biologically plausible that these routes can allow for lower immunological adverse reactions, while providing other benefits such as sterilizing immunity, as observed with intranasal vaccinations (34). Data on intranasal COVID-19 vaccines so far have not reported any adverse reactions, although the widespread usage of these vaccines is still a work in progress (36). Although relatively safe, there were reports of facial palsy with the administration of intranasal influenza vaccines (37). Intranasal vaccines against COVID-19 have not shown any such incidences to date (36).

## Characteristics of GBS associated with COVID-19 infection and vaccination

GBS, secondary to COVID-19, is majorly a post-infectious process with the onset of GBS-related symptoms separated by at least 2 weeks after COVID-19-related symptomatology (38). A few case reports, however, reported the onset of GBS in less than 2 weeks of COVID-19 infection (39). A recent case series identified viral RNA through advanced metagenomic sequencing in patients developing GBS within 4–15 days (about 2 weeks) of COVID-19 symptom onset, thus pointing toward a possible hypothesis of para-infectious processes (40). While a para-infectious process could be more likely secondary to direct viral invasion or the effect of inflammatory cytokine cascade on the peripheral nervous system, a post-infectious process could be an immunological phenomenon. A study that compared COVID-19-related early (para-infectious) and delayed (post-infectious) GBS showed no significant differences in clinical and electrodiagnostic profiles and serum and cerebrospinal fluid (CSF) cytokine levels (41).

The risk of GBS was higher in older men, with the majority being over 55 years of age (26, 42). The majority of COVID-19-associated GBS cases were sensory-motor type with a demyelinating pattern on electrodiagnostic studies (42, 43). Some studies have also reported higher mortality with COVID-19-related GBS as compared to other etiologies, which is independent of the mortality risk associated with COVID-19 infection (44). Several other rare variants of GBS, such as Miller Fisher Syndrome, pharyngeal cervical brachial variant, acute panautonomic neuropathy, and GBS with bifacial paralysis, have also been associated with COVID-19 infection (38, 42). Some studies have reported regional variations with the prevalence of more axonal variants from countries such as India and Iran and a higher proportion of autonomic dysfunction reported from the United States of America (43). While some initial studies did suggest a higher incidence of autonomic dysfunction and more severe illness in COVID-19-associated GBS (45), the subsequent studies did point toward no such difference. Overall, the clinical features, outcomes, and therapeutic strategies to treat COVID-19-related GBS resembled the cohort of the International Guillain Barre Syndrome Outcome Study (IGOS) (26).

Ever since the beginning of the pandemic, multiple variants of COVID-19 were prevalent across different timelines. Some of them were identified as variants of concern given their association with higher severity of COVID-19 disease. However, the current literature on COVID-19-associated GBS does not delineate the findings across different COVID variants. The studies published before 2021 (4, 20) would have more likely included alpha and beta variants, which showed that the demyelinating form of GBS was more common than other forms. Studies that evaluated cases across the pandemic, including most of the COVID-19 variants (26, 31), suggest that GBS, overall, had similar presentation and outcomes in both COVID-19-related and unrelated cases. The association of GBS following COVID-19 vaccines was based on temporality, with the mean duration of onset of GBS symptoms being 12–13 days (46). This duration also coincides with the maximal immune response to the vaccination. Interestingly, the onset of GBS was earlier (within 7 days) with mRNA vaccines, as compared to viral vector vaccines (para-infectious vs. immunological) (31). Most cases occurred after the first dose (46). Although currently there is limited understanding of how the response to vaccines is different among male and female subjects, some studies have suggested a higher incidence of GBS in men following adenovirus vector vaccination (30). The role of gender in mediating the immunological reaction to COVID-19 vaccinations leading to GBS is an area that warrants further investigation. Similar to classical GBS and GBS associated with COVID-19 infection, the most common subtype was demyelination with motor involvement, with older men being at higher risk (31, 46). The outcome and treatment response to classical therapies, such as intravenous immune globulin (IVIG), did not differ with COVID-19 infection or vaccine-unrelated GBS (31).

## Possible pathophysiological underpinnings of GBS associated with COVID-19 infection and vaccination

**Figure 1:**
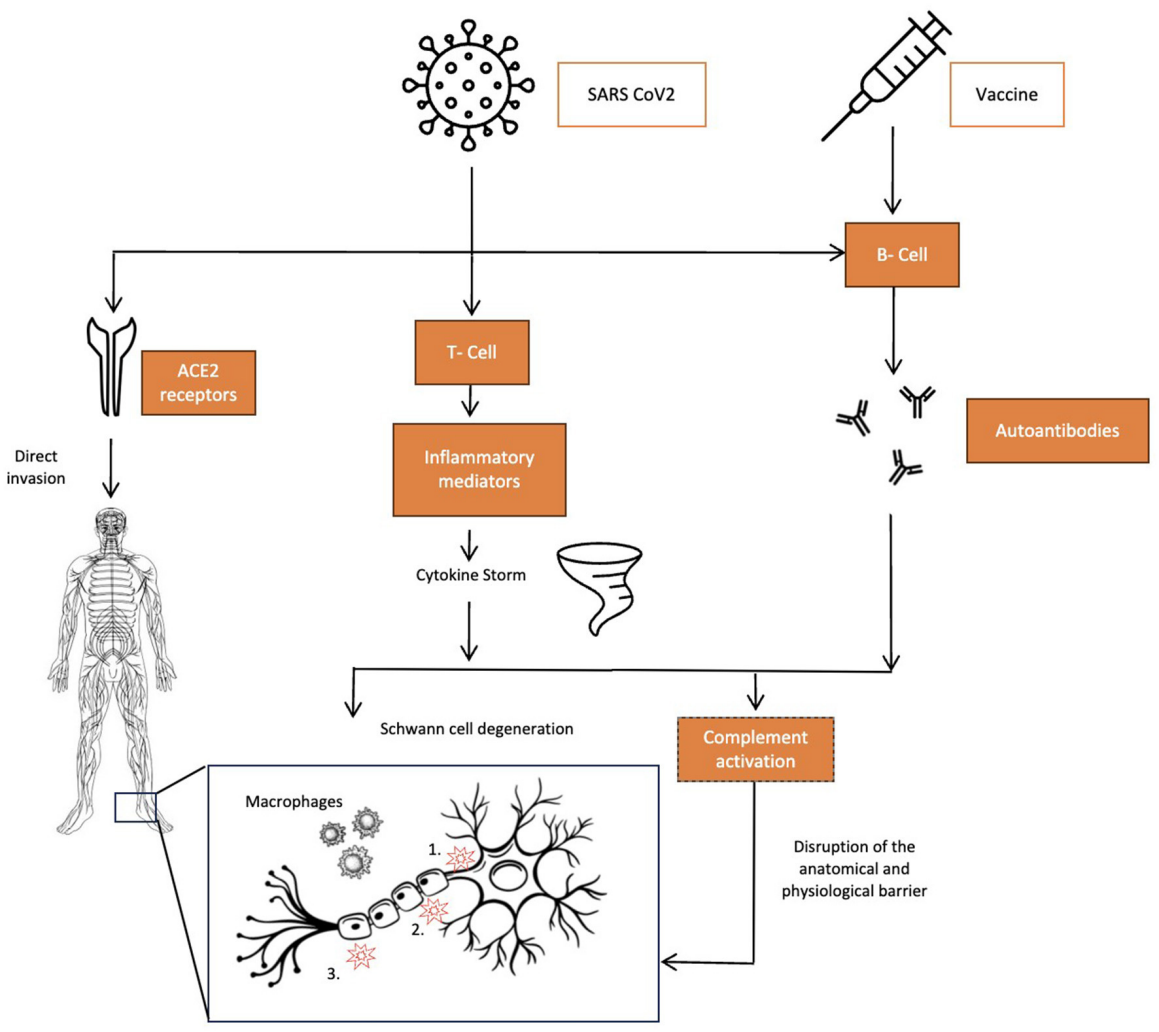
Pathophysiology of COVID-19 related GBS – schematic. The three pathophysiological mechanisms for GBS associated with COVID-19 infections. Left to right: direct invasion by the virus via ACE2 receptors in the central and peripheral nervous system causing direct toxicity in cases touted to be para-infectious forms of GBS. COVID-19 infections causing a cytokine storm due to markedly elevated levels of several proinflammatory mediators, the most prominent being TNF-α, which leads to complement activation, causing macrophage-lead myelin damage, and IL-17, which directly causes Schwann cell degeneration. Both SARS-CoV-2 and adenovirus vector vaccines cause humoral-mediated autoantibody formations such as GM1, GD1a, GD1b, GQ1b, and GT1b that target gangliosides on the nervous tissue, causing disruption of anatomical and physiological barriers with three main targets on nerves: 1. Axon; 2. nodes of Ranvier; and 3. Myelin.

The pathophysiology of GBS has been strongly related to autoimmunity. However, the exact biological mechanisms causing this association are still unclear. Based on the available literature, we can postulate various pathophysiological mechanisms that can provide potential therapeutic targets for GBS (Figure 1).

### Role of inflammation

Many studies have shown the role of active inflammatory mediators in the pathogenesis of GBS. TNF-α is elevated in GBS and is known to be correlated with disease severity (47). IL-17-mediated Schwan cell degeneration was studied in the literature (48). COVID-19 is a proinflammatory condition that causes cytokine release syndrome. Studies have shown elevated levels of cytokines in the CSF, suggesting a role of cytokine storm in the neurological manifestations of COVID-19 (49). Some authors suggest that GBS associated with COVID-19 in its para-infectious form is at least partly mediated by acute inflammation and cytokine release (50). It is evident that complement activation that triggers cytokine release has a role to play in pathogenesis as well. Some studies have targeted testing the role of complement inhibitors such as eculizumab in GBS, but the evidence of its safety is questionable (51).

### Active viral invasion

Active viral invasion is a less-tested and less-proven hypothesis in the pathogenesis of GBS in COVID-19. There are a few reports of identifying the SARS-CoV-2 virus in the CSF of patients with GBS from India and Brazil (52, 53). Another study identified the virus in the CSF of patients with GBS through metagenomic sequencing (40). Neuroinvasion could cause demyelination and axonal damage, leading to GBS manifestations. However, the para-infectious forms of GBS and many other studies have not been able to identify the COVID-19 virus in the CSF, bringing up questions against this hypothesis. Therefore, further investigation is needed to establish the role of neuroinvasion as one of the potential mechanisms of GBS in COVID-19 infection. The fact that GBS has a stronger association with adenovirus vector vaccines than the mRNA vaccines and the possible role of neuroinvasion by adenovirus vectors leading to peripheral nervous system dysfunction cannot be completely eliminated.

### Autoimmunity

The autoimmune hypothesis is most widely studied in GBS associated with COVID-19. This is very well tested with other infectious triggers such as *C*. je*juni*. Molecular mimicry and immunological cross-reactivity serve as potential mediating mechanisms. There are reports of COVID-19-associated GBS with positive testing for anti-ganglioside antibodies including anti GM1, GD1a, GD1b, GQ1b, and GT1b (54). Disruption of the blood–nerve barrier with direct exposure of neural antigens to the immune system has been studied in the pathogenesis of GBS (55). Induction of the hyperactive immune response by COVID-19 infection through mechanisms such as the depletion of NK cells and the formation of extracellular neutrophil traps. This can, in turn, lead to manifestations of autoimmunological conditions such as GBS (56). COVID-19 virus also was found to have two immunologically active hexapeptides that resemble certain heat shock proteins in the human nervous system, supporting the hypothesis of molecular mimicry in the pathogenesis of GBS in COVID-19 infection (57). Vaccinations against COVID-19 mediate their effect by triggering the immune response against the spike protein of the virus that helps binding with the host cell. The host-mediated immunological response could trigger autoimmunity and antibody production against the targets on the peripheral nervous system, leading to GBS (Figure 1).

## Conclusion and future directions

COVID-19 is known to affect the nervous system with varied neurological complications. Despite some controversy, GBS is found to have an association with COVID-19 infection. We hypothesize pathological mechanisms, such as inflammation, active viral invasion, and autoimmunity, mediating the association of GBS with COVID-19. However, the exact pathophysiological mechanisms and causality of COVID-19 on GBS remain to be investigated. Additional studies are needed to understand the exact pathophysiological underpinnings that could help develop potential therapeutic targets. Although adenovirus vector vaccines can increase the risk of GBS, data on the efficacy of mRNA vaccines against COVID-19 are reassuring, and these vaccines have been shown to decrease the risk of GBS. This brings into light the potential role of adenovirus vectors in mediating peripheral nervous system dysfunction. Further studies to understand this association are needed as the utility of adenovirus vectors is being increasingly utilized in many other gene therapies. Large-scale epidemiological studies strongly support evidence in favor of vaccination against COVID-19 infections in preventing COVID-19 infection and reducing the overall risk of GBS.

## Author contributions

VV: Writing – original draft, Writing – review & editing. SR: Writing – original draft, Writing – review & editing. CP: Supervision, Writing – original draft, Writing – review & editing. XL: Supervision, Writing – original draft, Writing – review & editing.
